# Effects of resveratrol supplementation to equine sperm during semen cryopreservation

**DOI:** 10.1590/1984-3143-AR2025-0107

**Published:** 2026-05-25

**Authors:** Natália de Castro Alves, Marina Morra Freitas, Jade Raquel Dias Farias, Cesar Lopes Horta, José de Oliveira Carvalho, Olindo Assis Martins-Filho, Márcio Sobreira Silva Araújo, Ângela Quintão Lana, Guilherme Mattos Jardim Costa, Fernanda Radicchi Lobato de Almeida, Erica Azevedo Costa, Monique de Albuquerque Lagares

**Affiliations:** 1 Escola de Veterinária, Universidade Federal de Minas Gerais – UFMG, Belo Horizonte, MG, Brasil; 2 Faculdade de Medicina Veterinária, Universidade Federal do Espírito Santo – UFES, Alegre, ES, Brasil; 3 Laboratório de Diagnóstico e Monitoramento de Biomarcadores, Centro de Pesquisa René Rachou, Fundação Oswaldo Cruz – Fiocruz, Belo Horizonte, MG, Brasil; 4 Instituto de Ciências Biológicas, Universidade Federal de Minas Gerais – UFMG, Belo Horizonte, MG, Brasil

**Keywords:** antioxidant, reproduction, cryopreservation, semen, stallion

## Abstract

During equine semen cryopreservation, most of the seminal plasma is removed, making sperm more susceptible to oxidative stress. This study evaluated the effect of resveratrol supplementation on the quality of frozen-thawed equine sperm. Semen from ten stallions was frozen using a control INRA 96 extender and extenders supplemented with 5-, 10-, 100-, and 150- µM resveratrol. Post-thaw evaluations included motility, kinematic parameters, morphology, plasma and acrosomal membrane integrity, mitochondrial potential, lipid peroxidation, nitrite, hydrogen peroxide, malondialdehyde levels, total reactive oxygen species (ROS), chromatin protamine deficiency, chromatin condensation, sperm binding to bovine oviduct explants, and gene expression of apoptosis-related genes such as B cell lymphoma 2 (BCL2) and BCL2-associated X (BAX), mitochondrial ROS modulator 1 associated with mitochondrial ROS production (ROMO1), sperm acrosome-associated 3 related with sperm binding capacity to the zona pellucida (SPACA3), and DNA damage repair gene 8-oxoguanine DNA glycosylase 1 (OGG1). Data were analyzed by ANOVA and Tukey test (*P* < 0.05). The 10 µM resveratrol treatment significantly increased sperm motility, mitochondrial activity, SPACA3 expression, and the number of sperm bound to oviduct explants compared to the control. Additionally, 10 µM resveratrol reduced total ROS, ROMO1, and BAX gene expression, indicating reduced oxidative stress and apoptosis. In conclusion, 10 µM resveratrol supplementation improved sperm metabolic activity, enhanced oviduct binding capacity, and demonstrated antioxidant and anti-apoptotic effects. Thus, incorporating resveratrol into freezing extenders could be a promising strategy to improve the fertilizing capacity of equine sperm in artificial insemination programs.

## Introduction

Cryopreservation is commonly used for long-term preservation of sperm from domestic animals and has undergone significant advances in recent years for species such as cattle, horses, pigs, and sheep (Yánez-Ortiz et al., 2022). However, the semen cryopreservation process causes various damages to sperm due to temperature changes and oxidative damage, compromising cell viability, motility, and longevity (Roca et al., 2013; Jakop et al., 2023). Semen has a limited antioxidant defense system, including glutathione peroxidase (GPx), superoxide dismutase (SOD), and catalase. Additionally, the high concentration of polyunsaturated fatty acids in sperm membranes let them more susceptible to oxidative stress inducing damage during freezing and thawing (Asadi et al., 2017; Xu et al., 2025).

Antioxidants, such as resveratrol, have been added to semen extenders to minimize oxidative stress during the cryopreservation process (Zhu et al., 2023). It has improved post-thaw semen quality in bulls (Bucak et al., 2015), stallions (Nouri et al., 2018; Du et al., 2025), dogs (Bang et al., 2021), and boars (Estrada et al., 2014; Kaeoket and Chanapiwat, 2023). However, to date the gene expression and the number of equine sperm bound to bovine oviduct explants of frozen equine sperm with resveratrol has not been evaluated.

Resveratrol (3,5,4'-trihydroxystilbene) is a polyphenol that belongs to dietary stilbenes, a class of compounds exhibiting significant biological activities of medicinal interest. This compound is one of the most well known and investigated polyphenols found in nature, produced by more than 70 different types of plants, and is present in red wine and various botanical extracts (Kozyra and Madalińska, 2025). In somatic and germ cells, resveratrol acts as a potent antioxidant by reducing mitochondrial reactive oxygen species (ROS) production, scavenging free radicals and inhibiting lipid peroxidation (Zhu et al., 2023; Xu et al., 2025). Accordingly, resveratrol supplementation during sperm cryopreservation has been associated with improved mitochondrial activity and reduced oxidative stress in different domestic species (Kaeoket and Chanapiwat, 2023; Du et al., 2025).

Equine exhibit variable fertility rate with artificial insemination, particularly with cryopreserved semen. Thus, strategies to maximize the fertilizing capacity of frozen/thawed equine sperm is crucial to the reproductive success. Therefore, the aim of this study was to evaluate whether the addition of resveratrol to semen freezing extenders improves the quality of equine frozen-thawed sperm.

## Methods

All experimental procedures were conducted in accordance with the ethical principles and animal welfare standards of Brazil for the use and care of animals in research. These procedures were approved by the Animal Ethics Committee “Comissão de Ética no Uso de Animais” (CEUA) of the Federal University of Minas Gerais (UFMG), protocol number 394/2017.

### Semen collection and evaluation

Two ejaculates were collected from 10 clinically healthy Mangalarga Marchador stallions (*n* = 10), aged between 4 and 9 years, located in Minas Gerais, Brazil, during the months march, october, november, and december. The stallions were selected based on andrological examination and reproductive history. After collection, semen was evaluated for sperm motility and vigor using a bright-field microscope (100x magnification). Only ejaculates with progressive motility equal to or greater than 50% and vigor equal to or greater than 3 were used. Sperm concentration per milliliter was calculated using a hemocytometer. Sperm morphology was evaluated using the wet preparation technique (Mies, 1975). Semen was preserved in a buffered formalin saline solution, and 200 sperm per sample were analyzed using a phase-contrast microscopy (x 1000). Only ejaculates with at least 70% or greater of normal morphologically sperm were used (CBRA, 2013).

### Semen freezing

The selected ejaculates were diluted in a 1:1 ratio with Kenney's extender (Kenney et al., 1975) and centrifuged (450 × g, 10 min). The supernatant was discarded, leaving 10% of the total volume. After homogenization, the semen was resuspended in INRA 96 freezing medium containing 2.5% glycerol and 2% supernatant of the centrifuged egg yolk (600 × g, 20 min, Pillet et al., 2008) to achieve a concentration of 100 × 10^6^ sperm/mL. Five treatments were performed: 0 (Control), 5, 10, 100, and 150 µM resveratrol. The semen was packaged in 0.5 mL straws and cooled for 1.5 h in a Styrofoam container (0.27 °C/min) to 5 °C. The straws were then placed 2.5 cm above liquid nitrogen, and after 20 min, they were immersed in it and stored in liquid nitrogen tanks (−196 °C).

### Post-thaw sperm evaluation

The straws were thawed at 37 °C and semen samples were analyzed including motility and kinematics, morphology, membrane functionality and integrity, rate of spontaneous acrosome reaction, lipid peroxidation, mitochondrial membrane potential, DNA fragmentation, nitrite, hydrogen peroxide, total reactive oxygen species, malondialdehyde concentrations, number of sperm bound to oviduct explants, and gene expression, as described following.

#### Computer-Assisted Sperm Analysis (CASA)

Sperm motility evaluation was performed thawing one straw of each treatment (37 °C, 30 s). A 10 µL aliquot of semen samples was placed on a Makler chamber (10 µm depth) and evaluated using a computer-assisted sperm analysis system (CASA, Sperm Class Analyzer, SCA® 2005 VS 4.0.0 Microptik S.L., Barcelona, Spain). Samples were assessed for total motility (TM%), progressive motility (PM%), curvilinear velocity (VCL-µm/s), average path velocity (VAP-µm/s), straight-line velocity (VSL-µm/s), amplitude of lateral head displacement (ALH-µm), beat-cross frequency (BCF-Hz), straightness (STR-%), linearity (LIN-%), and wobble (WOB-%). Five fields per sample were analyzed. CASA settings were capture rate of 25 images per second; optics: Ph-; chamber: Makler; scale: obj10x, particle area (in μm^2^) 4 < area < 75; curvilinear velocity (VCL): 10 < slow 45 < medium < 90 < fast µm/s; progressive motility > 75% straightness (STR), circular < 50% linearity (LIN).

#### Sperm morphology

Sperm morphology was evaluated using a wet preparation technique (Mies, 1975), where the semen samples were preserved in buffered formalin saline solution, and 100 sperm were assessed using a phase-contrast microscopy (x 1000).

#### Plasma membrane functionality evaluation

The percentage of sperm with functional plasma membrane (PM) was evaluated using the hypoosmotic swelling test (HOST) with distilled water (Lomeo and Giambersio, 1991), modified by Lagares et al. (2000) at a 1:2 dilution (semen: distilled water). After semen samples thawing, a 100 µL aliquot was added to 200 µL of distilled water at 37 °C. After 5 min incubation, 200 sperm were analyzed using a phase-contrast microscopy (x 400). Tail curling sperm was considered positively react to the HOST. The final percentage of HOST+ sperm was obtained after subtracting the percentage of curled tail sperm observed in the morphological examination.

#### Plasma and acrosomal membrane integrity evaluation

The integrity of plasma (PM) and acrosomal membranes (AM) was evaluated using a flow cytometry with Propidium Iodide (PI, Sigma P4170, Sigma Chemical St. Louis, MO, USA) and Fluorescein Isothiocyanate-Peanut Agglutinin (FITC-PNA, Sigma L7381, Sigma Chemical St. Louis, MO, USA) fluorescent probes. The thawed semen samples were diluted 1:20 in a phosphate-buffered saline (PBS, 250 μl semen: 5 mL PBS). A 200 μL aliquot of this suspension was stained with FITC-PNA (1.125 μg/mL) and incubated (10 min, 37 °C). Samples were then diluted by adding 1 mL PBS, stained with PI (1.5 mM), and incubated for 10 min at room temperature. The samples were analyzed using a flow cytometer (FACScalibur6: BD Biosciences, San Jose, CA, USA). Ten thousand events per sample, at a rate of 500 cells per second, were analyzed. The following sperm categories were observed: PI+/PNA^+^: sperm with non-intact PM and AM, PI+/PNA-: sperm with non-intact PM and intact AM, PI-/PNA-: sperm with intact PM and AM, PI-/PNA^+^: sperm with intact PM and non-intact AM. Sperm with spontaneous acrosome reaction were considered those with PI-/PNA^+^, and sperm with intact PM without spontaneous acrosome reaction were those with PI-/PNA-.

#### Mitochondrial membrane potential assessment

Mitochondrial membrane potential was assessed using flow cytometry with the fluorescent probes Propidium Iodide (PI, Sigma P4170, Sigma Chemical St. Louis, MO, USA) and MitoTracker Green FM (Invitrogen M7514, Massachusetts, USA). A 10 million sperm aliquot was diluted in 2 mL of PBS and centrifuged (12 min, 500 × g). The supernatant was discarded, and the samples were resuspended in 200 µL of PBS. A volume of 0.5 µL of the pre-warmed (37 °C) stock solution of MitoTracker Green FM (final concentration 20 nM) was added to the samples and incubated in the dark (37 °C, 15 min). Subsequently, the samples were diluted with 1 mL PBS, stained with PI (1.5 mM), and incubated (37 °C, 5 min). After that, the sperm samples were washed twice with 2 mL PBS in the tubes and centrifuged (12 min, 500 × g). The supernatant was discarded and the sperm were resuspended in 1 mL PBS. The samples were analyzed using a flow cytometer (FACScalibur6: BD Biosciences, San Jose, CA, USA). Ten thousand events per sample were analyzed at a rate of 500 cells per second.

#### Sperm lipid peroxidation assessment

Sperm lipid peroxidation was evaluated using the fluorescent probe C11-BODIPY581/591 (Life Technologies Ltd., Grand Island, NY, USA) and assessed by flow cytometry according to Partyka et al. (2011). A volume of 20 µL semen was washed by centrifugation (500 × g, 5 min) and resuspended in 160 µL PBS. Then, 20 µL of C11-BODIPY581/591 (5 µM) was added and incubated in the dark (37 °C, 30 min). The samples were then washed by centrifugation (500 × g, 5 min) and the pellet was resuspended in 500 µL PBS. The samples were analyzed with a flow cytometer (FACScalibur6: BD Biosciences, San Jose, CA, USA). Ten thousand events per sample were analyzed at a rate of 500 cells per second. The positive control was performed by incubating a sperm sample in hydrogen peroxide (500 µM) at 37 °C for 60 min.

#### Measurement of nitrite (NO^2-^) concentration

The measurement of nitrogen reactive species concentration was performed by measuring the levels of nitrite (NO^2-^) in μM/μg of protein. Nitrite is one of the two primary, stable, and non-volatile products of NO decomposition, and its measurement using spectrophotometry with the Griess reagent (Green, 1997) is a way to investigate nitric oxide formation. The Griess Reagent System is based on the chemical diazotization reaction that uses 2% (w/v) sulfanilamide and 0.2% (w/v) N-(1-naphthylethylenediamine dihydrochloride (NEED) under acidic conditions (5% (v/v) phosphoric acid). The detection limit is nitrite of 2.5 μm (125 pmol) (in ultrapure, deionized distilled water). The absorbance of the samples was determined at 560 nm. Griess reagent was prepared at the time of analysis and kept protected from light throughout the experiment. In a 96-well microplate, 50μL of thawed semen sample (37 °C, 30 s) from each treatment and 50μL of Griess reagent were added, and for the blank treatment, 50 μL of distilled water and 50μL of Griess reagent were added in triplicate. The microplate was read in a spectrophotometer at 560 nm.

#### Measurement of hydrogen peroxide (H_2_O_2_) concentration

The measurement of hydrogen reactive species concentration (H_2_O_2_) was performed by measuring hydrogen peroxide (H_2_O_2_) levels in μM/μg of protein using spectrophotometry. H_2_O_2_ concentration was measured by the method modified by FOX-2 (Nourooz-Zadeh et al., 1994). This technique is possible by the oxidation of ferrous ions (Fe^2+^) to ferric ions (Fe^3+^) under acidic conditions by lipid hydroperoxides. The indicator used is xylene orange, which reacts with Fe3+ ions producing a blue-purple chromophore with an extinction coefficient of 4.3x10^4^ M-1cm-1, detected in the spectrophotometer reading at 560 nm. H_2_O_2_ concentration was determined in the samples according to the molar extinction coefficient of H_2_O_2_. Therefore: Aλ=Ԑλ.C, where Aλ=absorbance at 560nm; Ԑλ=molar extinction coefficient of the chromophore; C=concentration of hydroperoxides (mol/mL).

#### Measurement of malondialdehyde concentration

To determine the rate of lipid peroxidation of post-thawed sperm, another technique was used to measure the concentration of malondialdehyde (MDA), using thiobarbituric acid (TBA) based on the method described by Buege and Aust (1978). Malondialdehyde levels were measured after adding 250 µL of semen to 500 µL of TBA reagent (15% trichloroacetic acid, 0.25N hydrochloric acid, and 0.375% thiobarbituric acid) and 1% (v/v) BHT 50mM, and this solution was boiled (100 °C) for 15 min. Subsequently, the samples were cooled on ice flakes and centrifuged (1,200 × g, 15 min). The supernatant was removed and measured in the spectrophotometer with absorbance at 532 nm. TBARS quantification in the samples was performed using a standard curve of 1 to 20nMol of MDA/mL. The results were expressed in nMol of TBARS/mL.

#### Measurement of total levels of reactive oxygen species (ROS)

The cellular level of ROS was evaluated using the 2',7'-dichlorofluorescin diacetate (DCFDA) kit (ab113851, Abcam, Cambridge, England). The reactive oxygen species (ROS) assay kit uses the permeable DCFDA reagent (2',7'-dichlorofluorescin diacetate), a fluorogenic dye that measures the activity of hydroxyl, peroxyl, and other ROS within the cell. After diffusion into the cell, DCFDA is deacetylated by cellular esterases into a non-fluorescent compound, which is subsequently oxidized by ROS into 2',7'-dichlorofluorescein (DCF). DCF is a highly fluorescent compound that can be detected by fluorescence spectroscopy. For analysis, samples containing 1.5 × 10^6^ sperm were washed in PBS and incubated at 37 °C for 30 min with 1 mL of buffer solution containing 20 µM DCFDA. Subsequently, the sperm samples were washed with 1 mL of 1X buffer solution and resuspended in 500 µL of 1X supplemented buffer solution. Finally, 50 µL of the sperm suspension from each treatment was plated in triplicate in wells of a dark-bottom microplate. Fluorescence intensity was detected using the Cytation 5 microplate reader (Biotek, Santa Clara, CA, USA) at an excitation wavelength of 485 nm and emission wavelength of 535 nm. The intensity of their fluorescence was measured, the sperm with elevated ROS levels were stained green, while those with lower ROS levels exhibited reduced fluorescence. For the positive control, sperm were treated with 50 µM tert-butyl hydroperoxide (tbHP) for 3 h at 37 °C.

#### Evaluation of chromatin protamination deficiency in sperm using Chromomycin A3 (CMA3)

To assess sperm chromatin protamination, the fluorescent dye CMA3 (Sigma, St. Louis, MO, USA) was used (Simões et al., 2009). Thawed semen samples (37 °C, 30 s.) were diluted to 2x10^6 sperm in 200 μL of McIlvaine's buffer (17 mM citric acid, 164 mM Na2HPO4, and 10mM MgCl26H2O; pH 7.0) and centrifuged (500 × g, 5 min.). After discarding the supernatant, the sample was suspended in 200 μL of Carnoy's solution (3 parts methanol and 1 part acetic acid) and kept at 4 °C for 5 min. After two washes with PBS, the samples were incubated for 60 min at room temperature with CMA3 staining solution (0.25mg/mL of CMA3 in McIlvaine's buffer). Subsequently, the samples were washed and resuspended in 1 mL PBS. Then, the samples were analyzed in a flow cytometer (FAC Scalibur 6 BD Biosciences, San Jose, CA, USA). Ten thousand events at a rate of 500 cells per sec, were analyzed per sample. For the positive control, 2x10^6 sperm were fixed in 7.5 mL of chlorine-free Carnoy's solution (3 methanol: and 1 acetic acid), incubated for 10 min at room temperature, and centrifuged at 4 °C (3.074 × g, 15 min). After discarding the supernatant, the sample was washed with 2 mL of PBS, and the pellet was resuspended and incubated with 0.6 mL of PBS and 0.1% Triton (15 min/37 °C). The permeabilized sample was centrifuged at 4 °C (3.074 × g, 15 min), the supernatant was discarded, and the pellet was resuspended and incubated in 0.6 mL of 1mM NaCl and 5mM dithiothreitol (DTT) for 1 h at room temperature. The DTT-treated sample was centrifuged at 4 °C (3.074 g, 15 min), the supernatant was discarded, and the pellet was resuspended in 90 µL of PBS. The positive control was incubated for 60 min at room temperature with the CMA3 staining solution (0.25mg/mL of CMA3 in McIlvaine's buffer). After 60 min, the sample was washed and resuspended in 1 mL of PBS. Then, the sample was analyzed in a flow cytometer (FACScalibur6: BD Biosciences, San Jose, CA, USA).

#### Chromatin condensation with Toluidine Blue

The sperm chromatin condensation was prepared with smears of thawed semen. The smears were fixed in a ethanol solution and acetic acid (3:1) for one minute and then in 70% ethanol for 3 min. The smears were air-dried and hydrolyzed for 15 min in 4N hydrochloric acid. They were then washed in distilled water and air-dried at room temperature. A 10 μl drop of 0.025% toluidine blue in McIlvaine's buffer was added, covered with a coverslip, and left to stain for 3 min. After that 500 sperm per slide were evaluated under microscope (x1000) and classified as with compact chromatin (lightly stained head) or decondensed chromatin (dark blue or violet-stained head).

#### Number of equine sperm bound to bovine oviduct explants

Dissected oviducts from ovaries without a corpus luteum of cows slaughtered in an Abatoir were used. The isthmus region was pressed on a Petri dish containing TCM 199 culture medium + 10% FBS + 0.1 mg/mL streptomycin + 100 IU/mL penicillin. The oviduct cells were aspirated with a 30 × 0.8 mm needle attached to a 5 mL syringe and transferred to a 14 mL Falcon tube for sedimentation. This procedure was repeated three times to disaggregate the oviduct cells, and then they were transferred to drops with TCM 199 medium with Hepes, 10% FBS, 0.1 mg/mL streptomycin, and 100 IU/mL penicillin for reaggregation and explant formation for 24 h of incubation at 38.5 °C with 5% CO_2_. An aliquot of semen from each treatment with 1x10^6^ sperm/mL was distributed in 80 µL drops containing 25 explants/treatment/stallion, and the sperm-explant incubation was carried out for 30 min. Three washes with TCM 99 medium were performed to remove sperm not bound to the explants. The explants were placed between a slide and a coverslip, and the number of sperm bound to the perimeter of the explants was calculated using Image J software (Version 1.52a).

#### 2.3.14. Gene expression Real-time quantitative polymerase chain reaction (RT-qPCR)

Gene Expression RT-qPCR was used to analyze the expression of genes related to apoptosis, such as B cell lymphoma 2 (BCL2) and BCL2-associated X (BAX), mitochondrial ROS modulator 1 (ROMO1), sperm-associated antigen 3 (SPACA3), and oxidative damage repair DNA glycosylase 1 (OGG1).

Total RNA was extracted from post-thawed semen samples cryopreserved with varying niacin concentrations or without niacin (control) using the QIAamp Viral RNA Mini Kit, following the manufacturer’s instructions. RNA quantification was performed with the NanoDrop Lite Plus (Thermo Fisher Scientific, Massachusetts, USA) and standardized to a concentration of 160 ng/reaction. Complementary DNA (cDNA) synthesis was carried out using the Oligo dT_18_ Primer (Thermo Fisher Scientific, Massachusetts, USA) as per the manufacturer’s protocol. Transcript expression levels were evaluated by RT-qPCR using the qPCR Green Master Kit (Cellco Biotech, São Paulo, Brazil), with primers listed in Table 1. The qRT-PCR primer pairs for the OGG1, ROMO1, and SPACA3 genes were designed in this study using Primer-BLAST from NCBI, based on the reference sequences listed in Table 1. Gene expression was quantified relative to the internal β-actin gene using the equation R=2 ^– [∆Ct sample −∆Ct control]^.

**Table 1:** Primer sequence used for gene expression analysis of post-thaw equine sperm.

Gene	Primer sequence (5′ –3′)	Product size (bp)	NCBI Accession Number (Reference)
β-actin	F: CCAGCACGATGAAGATCAAG	88	AF035774 (Pérez Rico et al., 2014)
	R: GTGGACAATGAGGCCAGAAT		
BAX	F: TTTGCTTCAGGGTTTCATCC	162	XM_001489207.1 (Leon et al., 2013)
	R: ATCCTCTGCAGCTCCATGTT		
BCL2	F: GAGACCCCCAGTGCCATCAA	146	XM_001499714.1 (Leon et al., 2013)
	R: GGGATGTCAGGTCGCTGAAT		
OGG1	F: AACAACAACATTGCCCGCAT	100	XM_023620020 (This study)
	R: GGAAGCCATGGTAGGTGAC		
ROMO1	F: TTCAGTCCTCCGCTACGGG	98	XM_005604601.3 (This study)
	R: GTCTCACACAGAACGCAAGG		
SPACA3	F:AACACAGCTGCCGTGGAC	76	XM_023653031 (This study)
	R: ACCACTTCCGGCTGTTGA		

BAX, BCL2-associated X; BCL2, B cell lymphoma 2; OGG1, 8-oxoguanine DNA glycosylase 1; ROMO1, Reactive Oxygen Species Modulator 1; SPACA3, Sperm Acrosome Associated 3.

#### Statistical analysis

The experimental design used was randomized complete blocks, considering the stallion as the block. Statistical analysis of motility characteristics, measurement of oxidants, DNA fragmentation, membrane integrity and functionality, spermatic lipid peroxidation, and binding rate of equine sperm to bovine oviduct explants was performed through analysis of variance (ANOVA) and compared with Tukey's test. Comparison of gene expression was performed using independent samples t-test. Data were analyzed using the R program, with a probability of *P* < 0.05 considered significant.

## Results

The mean sperm characteristics of two semen collections from 10 stallions were calculated. The ejaculates showed total motility (TM) ranging from 70 to 90%, progressive motility (PM) between 60 and 80%, and sperm vigor of 3 and 4. All characteristics were within the desirable range for semen cryopreservation according to the Manual for Andrological Examination and Animal Semen Evaluation of the Brazilian College of Animal Reproduction (CBRA, 2013). Sperm concentration ranged from 180 x10^6^ to 522 x10^6^ sperm/mL and the total number of sperm in the ejaculate from 5.2 x10^9^ to 18.7 x10^9^. The percentage of morphologically normal sperm in the ejaculate ranged from 75% to 85%.

The addition of 10 µM resveratrol significantly increased the percentage of sperm with TM compared to the control when evaluated by the CASA system (Table 2, *P* < 0.05). The treatments with 5-, 10- and 100- µM Resveratrol showed similar TM (*P* > 0.05). The other sperm motility and kinematics variables did not differ among treatments (Table 2, *P* > 0.05).

**Table 2:** Post-thawed sperm motility, kinematic parameters evaluated by CASA system and percentage of normal morphologically sperm in different treatments with different concentrations of Resveratrol (X ± SE).

Treat.	TM (%)	PM (%)	RAP (%)	VAP (µm/s)	VSL (µm/s)	VCL (µm/s)	BCF (Hz)	STR (%)	LIN (%)	WOB (%)	ALH (µm)	Normal (%)
0	41.3 ± 5.0^b^	10.6 ± 1.4	10.7 ± 0.2	18.9± 1.4	13.4± 1.2	28.5± 1.7	9.0± 0.7	70.5± 1.9	46.8± 2.6	66.2± 2.2	2.2± 0.1	76.6± 1.8
5	51.3± 5.9^ab^	10.7± 1.7	10.9± 0.3	19.2± 1.3	13.6± 1.1	28.5± 1.7	8.4± 0.6	70.2± 1.7	47.4± 2.4	67.1± 2.1	2.2± 0.1	76.70± 1.8
10	56.2± 5.8^a^	10.9± 1.7	11.3± 0.3	19.8± 1.1	14.3± 0.9	29.1± 1.7	8.4± 0.5	72.9± 1.7	49.5± 2.5	68.4± 2.1	2.2± 0.1	76.5± 1.8
100	51.2± 5.8^ab^	10.9± 1.7	11.4± 0.3	18.8± 1.3	13.4± 1.3	28.8± 1.7	8.7± 0.6	70.9± 1.7	46.9± 2.5	65.7± 2.1	2.3± 0.1	76.7± 1.8
150	44.5± 5.9^b^	9.8± 1.7	11.2± 0.3	18.5± 1.2	13.3± 1.1	27.9± 1.9	8.2± 0.6	71.2± 1.7	47.5± 2.4	66.4± 2.1	2.2± 0.1	75.9± 1.8

ab Values with different superscripts between lines differ statistically (*P* < 0.05), Treat: treatments, 0 = INRA 96 (control), 5, 10, 100, 150 = 5, 10, 100,150 µM Resveratrol; TM: % total motility; PM: % progressive motility; VAP: average path velocity; VSL: straight-line velocity; VCL: curvilinear velocity; BCF: beat-cross frequency; STR: straightness; LIN: linearity; WOB: wobble index; ALH: amplitude of lateral head displacement.

The percentage of normal morphologically sperm and sperm defect did not differ statistically among the treatments (Table 2, *P* > 0.05).

No difference regarding the percentage of sperm with functional plasma membrane (HOST+) and intact (PI-) and spontaneous acrosome reaction (PI-/PNA^+^) were observed between the treatments (Table 3, *P* > 0.05).

**Table 3:** Percentage of sperm with functional membrane (HOST+), intact plasma membrane (PM), spontaneous acrosome reaction (RA), sperm DNA protamination deficiency and decondensation with resveratrol addition (X ± SE).

Treat.	% HOST +	% intact PM	% RA	% DNA protamination deficiency	% Chromatin decondensation
0	42.4 ± 3.5	29.7 ± 2.5	0.20 ± 0.1	5.0 ± 0.9	3.4 ± 0.1
5	44.9 ± 4.5	30.9 ± 2.5	0.29 ± 0.1	3.8 ± 0.5	2.4 ± 0.1
10	50.2 ± 4.6	28.3 ± 2.4	0.19 ± 0.1	4.3 ± 0.5	3.3 ± 0.1
100	52.3 ± 4.5	27.3 ± 2.5	0.21 ± 0.1	5.1 ± 0.7	4.1 ± 0.1
150	45.2 ± 5.6	29.7 ± 2.4	0.24 ± 0.1	4.7 ± 0.6	3.0 ± 0.1

Treat: treatments, 0 = INRA 96 (control), 5, 10, 100, 150 = 5, 10, 100, 150 µM Resveratrol. There was no statistical difference between the values within rows and columns (*P* > 0.05).

There were no statistical differences on the percentage of sperm DNA protamination deficiency and decondensation with different resveratrol concentrations using CMA3 and Toluidine Blue (Table 3, *P* > 0.05).

**Figure 1:**
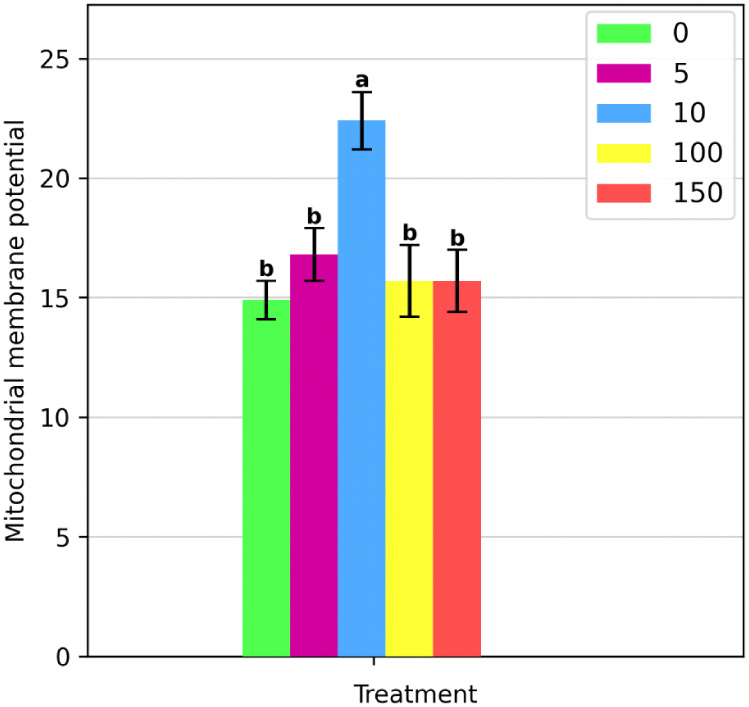
Percentage of sperm with mitochondrial membrane potential (Mitotracker+) and intact plasma membrane (PI-) with the addition of different concentrations of resveratrol (X ± SE). ^ab^Values with different letters between columns differ statistically (*P* < 0.05). 0 = INRA 96 (control), 5, 10, 100, 150 = 5, 10, 100, 150 µM Resveratrol.

The addition of 10 µM resveratrol significantly increased the percentage of sperm showing mitochondrial membrane potential (Mitotracker+) and intact plasma membrane (PI-, Figure 1, *P* < 0.05).

The addition of different concentrations of resveratrol to the freezing extender did not affect the sperm percentage with lipid peroxidation (BODIPY+), and the concentrations of NO^2-^, H_2_O_2_, and malondialdehyde produced by the sperm among the treatments (Table 4, *P* > 0.05). However, the addition of 10 µM resveratrol significantly decreased the sperm fluorescence intensity of total reactive oxygen species (Table 4, *P* < 0.05), which means that resveratrol decreases the total reactive oxygen/nitrogen species in the cryopreserved sperm.

**Table 4:** Sperm percentage with lipid peroxidation (BODIPY+), H_2_O_2_ and NO^2- (^µM/µg protein), and malondialdehyde (nM/mL of TBARS/mL) concentration and fluorescence intensity (AU) of total ROS (X ± SE).

Treat.	Lipid peroxidation	H_2_O_2_	NO^2-^	Malondialdehyde	Total ROS
	%	µM/ug protein		nM/mL TBARS/mL	AU
0	19.3 ± 0.8	26.5 ±1.4	9.6 ± 0.8	7.9 ± 0.7	3735 ± 380 ^a^
5	18.9 ± 0.2	26.2 ± 1.2	9.8 ± 0.9	6.6 ± 0.8	3160 ± 371 ^a^
10	16.8 ± 0.3	24.1 ± 1.1	9.9 ± 0.8	5.2 ± 0.7	1887 ± 203 ^b^
100	19.3 ± 0.2	24.3 ± 1.2	9.5 ± 0.9	5.4 ± 0.8	3437 ± 440 ^a^
150	18.7 ± 0.2	24.9 ± 1.3	9.4 ± 0.8	6.6 ± 0.8	3433 ± 407 ^a^

ab Values with different superscripts between lines differ statistically (*P* < 0.05). Treat: treatments, 0 = INRA 96 (control), 5, 10, 100, 150 = 5, 10, 100, 150 µM Resveratrol.

**Figure 2:**
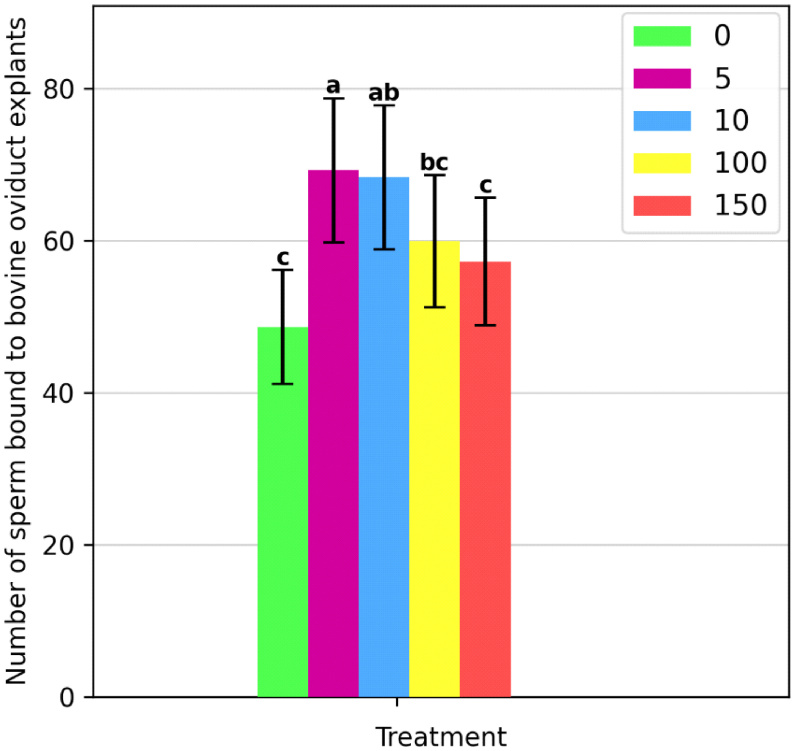
Number of sperm bound to bovine oviduct explants with the addition of different concentrations of resveratrol (X ± SE). ^abc^Values with different letters between columns differ statistically (*P* < 0.05). 0 = INRA 96 (control), 5, 10, 100, 150 = 5, 10, 100, 150 µM Resveratrol.

The addition of 5 and 10 µM resveratrol to the semen freezing extender significantly increased the number of sperm bound to bovine oviduct explants compared to the control and 150 µM (Figure 2, *P* < 0.05). The 100 µM resveratrol showed similar number of sperm bound to bovine oviduct explants in comparison to 10 and 150 µM.

**Figure 3:**
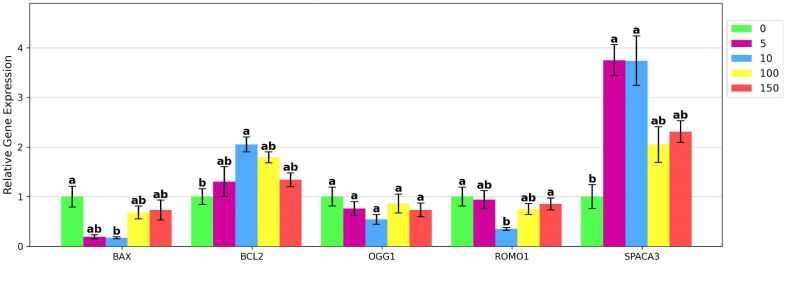
BAX: BCL2-associated X protein, BCL2: B cell lymphoma 2, OGG1: 8-oxoguanine DNA glycosylase 1, ROMO1: reactive oxygen species modulator 1, SPACA3: sperm acrosome associated 3. ^ab^Values with different letters between columns differ statistically (*P* < 0.05). 0 = INRA 96 (control), 5, 10, 100, 150 = 5, 10, 100, 150 µM Resveratrol.

The semen samples supplemented with 10 µM resveratrol showed a significantly increased gene expression level of BCL2 and a reduced expression of BAX compared to the control group (*P* < 0.05, Figure 3). The expression level of the ROMO1 gene was significantly reduced in semen samples supplemented with 10 µM resveratrol compared to the control (*P* < 0.05, Figure 3). On the other hand, expression levels of OGG1 gene did not show statistical differences among the treatments (Figure 3, *P* > 0.05). Additionally, the expression levels of SPACA3 gene in post-thaw sperm were significantly higher in semen samples supplemented with 5 and 10 µM resveratrol compared to the control group (*P* < 0.05, Figure 3).

## Discussion

In the present study, the addition of 10 µM resveratrol to the equine semen freezing extender increased motility, mitochondrial activity, BCL2 gene expression, and decreased total ROS and ROMO1 and BAX gene expression. These results indicated that resveratrol protects sperm metabolism, has antioxidant and antiapoptotic actions during the cryopreservation process. Additionally, 5 and 10 µM resveratrol increased SPACA3 gene expression and the number of sperm bound to bovine oviduct explant cells.

In our study, increased levels of the anti-apoptotic gene BCL2 and reduced levels of the pro-apoptotic gene BAX indicated that resveratrol reduced apoptosis during cryopreservation. Beneficial effects of resveratrol on sperm were also reported in other species and with other concentrations. Resveratrol treatment reduced apoptosis in cryopreserved dog semen with 200 µM resveratrol (Bang et al., 2021) and post-thaw boar semen supplemented with 1mM (He et al., 2020) and 50 µM resveratrol (Kaeoket and Chanapiwat, 2023).

ROMO1 is a key gene for generating mitochondrial ROS production (Shin et al., 2013), and high levels of endogenous ROS created in the mitochondrial respiratory chain can cause random genetic alterations, mutations, and lead to apoptosis (Xu et al., 2025). In our study, after resveratrol supplementation, the gene expression level of ROMO1 was significantly reduced compared to the control. Our results suggested that resveratrol can protect mitochondria during sperm freezing- thawing process resulting in ROS reduction.

The sperm acrosome-associated gene 3 (SPACA3) conserves substrate binding sites for N-acetylglucosamine oligosaccharides present in the extracellular matrix around the plasma membrane. Leung et al. (2023) reported that SPACA3 gene is associated with sperm binding capacity to the zona pellucida. In the present study, increased SPACA3 expression with 5 and 10 µM resveratrol might be related to an increase of sperm binding capacity to the zona pellucida. In cryopreserved dog semen supplemented with 200 µM resveratrol reduced ROMO1 gene expression and increase SPACA3 expression (Bang et al., 2021)

Additionally, in the present work, 5 and 10 µM resveratrol increased the number of sperm bound to bovine oviduct explant cells. The formation of a sperm reservoir in the oviduct epithelium plays a key role in regulating sperm capacitation, prolonging sperm viability within the female reproductive tract, and synchronizing sperm release with ovulation, thereby contributing to fertilization efficiency (Yánez-Ortiz et al., 2022; Pollard et al., 2022). Using an oviduct explant model, it was demonstrated that stallion sperm binding to the epithelium could induce capacitation in vitro (Leemans et al., 2014). The number of sperm bound to the explant in coculture showed a positive correlation with fertility rates. Additionally, sperm from the higher fertility stallion group remained bound to the explant for a longer period compared to the average group, indicating that factors related to binding capacity are directly linked to fertility (Leung et al., 2023). In the present study, the addition of 5 and 10 µM resveratrol increased the number of sperm bound to bovine oviduct explant cells.

Resveratrol addition to semen extenders has been carried out in various species. In ovine semen, supplementation with 50 µM resveratrol during cryopreservation reduced lipid peroxidation, improved antioxidant enzyme activity, and enhanced post-thaw motility and membrane integrity (Zhu et al., 2023). In boar semen, the addition of 50 µM resveratrol to freezing extenders significantly improved post-thaw sperm quality, including motility, membrane stability, and oxidative balance (Kaeoket and Chanapiwat, 2023). In equine semen, supplementation with 40 µM resveratrol during cryopreservation increased post-thaw sperm quality and antioxidant capacity, corroborating the present findings (Du et al., 2025).

The addition of resveratrol to equine semen during cryopreservation has shown dose-dependent effects. In Mongolian horses, concentrations of 10–40 µM improved total and progressive motility, plasma membrane integrity, and post-thaw antioxidant capacity, whereas higher concentrations (≥80 µM) acted as pro-oxidants, impairing sperm function (Du et al., 2025). Similarly, studies in sheep showed that 50 µM resveratrol optimized motility, membrane integrity, and mitochondrial potential, while concentrations above 100 µM had deleterious effects (Zhu et al., 2023).

Nouri et al. (2018) selected Arab stallions with low sperm quality and added 5, 10, and 20 µM resveratrol in a milk-based freezing extender, supplemented with 4% egg yolk and 5% N-dimethylformamide. The concentration of 5 µM resveratrol did not influence the evaluated parameters, however, 20 µM had deleterious effects on total motility. Corroborating to the present study, positive results were obtained with 10 µM, on total and progressive motility, plasma membrane integrity, viability, mitochondrial membrane potential, ROS production, and DNA fragmentation (Nouri et al., 2018).

The sperm freezing and thawing process increases ROS production, which negatively affects sperm viability, motility, and fertilization capacity (Du et al., 2025). Sperm motility, viability, and mitochondrial activity are the functions most affected by ROS activity, which can lead to reduced fertility at the time of artificial insemination (Lecewicz et al., 2018. Extender supplementation with antioxidants can neutralize oxidative stress and protect sperm against the adverse effects of ROS (Li et al., 2023). This study indicated that resveratrol protected mitochondrial function and reduced total ROS production during the freezing process. Similar semen freezing results were reported in humans, where 0.1, 1, and 10 mM of resveratrol reduced post-thaw lipoperoxidation in both fertile and infertile men (Garcez et al., 2010).

Studies have shown that resveratrol activates the AMPK pathway in sperm. AMPK is a key kinase involved in regulating cellular redox state, altering metabolic pathways under stress conditions (Takeo et al., 2014). In human sperm, it has been demonstrated that AMP-activated protein kinases are present mainly throughout the flagellum and the post-equatorial region of the head (Nashtaei et al., 2017). Related to these findings, resveratrol supplementation increased AMPK activity and was beneficial to protect cryopreserved human sperm against oxidative stress improving its DNA integrity (Nashtaei et al., 2017). Similar effects have been reported in pigs and goats, where resveratrol promoted AMPK phosphorylation, reduced ROS production, and enhanced glutathione (GSH) levels as well as the activities of glutathione peroxidase (GPx), superoxide dismutase (SOD), and catalase (Zhu et al., 2018, 2019; Li et al., 2023).

AMPK has been identified in human and stallion sperm with a regulatory role in sperm motility and maintaining sperm quality during prolonged storage (Hurtado de Llera et al., 2012; Cordova et al., 2014). Moreover, AMPK activation has been shown to reduce ROS levels in chicken sperm cryopreservation, restore ATP levels, and strengthen the antioxidant system, leading to improved post-thaw sperm quality (Nguyen et al., 2015). Collectively, these findings indicate that resveratrol protects sperm from cryoinjury through both direct antioxidant activity and AMPK-mediated enhancement of endogenous defense mechanisms, ultimately improving post-thaw sperm function across species (Du et al., 2025; Li et al., 2023).

## Conclusion

Supplementation of 10 µM resveratrol to semen freezing extender improved equine sperm metabolism, has antioxidant action, reduced apoptosis and increased the number of sperm bound bovine oviduct explants. Thus, 10 µM resveratrol addition to freezing extender might be beneficial to increase the fertility of cryopreserved semen in equine artificial insemination programs.

## Data Availability

Research data is available in the repository of the Federal University of Minas Gerais (UFMG).
